# Molecular characterization and zoonotic potential of *Blastocystis* subtypes in domestic pigs and cattle from Hainan, a tropical island province in China

**DOI:** 10.1051/parasite/2025070

**Published:** 2025-12-05

**Authors:** Yun Zhang, Jiaqi Li, Xiuyi Lai, Yuan Wang, Xinhui Li, Guangxu Ren, Xingyue Yu, Yu Li, Rui Liu, Yu Qiang, Tingting Li, Yunfei Zhou, Sheng Lei, Yuexiao Wu, Wei Zhao, Gang Lu

**Affiliations:** 1 Key Laboratory of Tropical Translational Medicine of Ministry of Education, Hainan Medical University-The University of Hong Kong Joint Laboratory of Tropical Infectious Diseases, Department of Pathogenic Biology, School of Basic Medicine and Life Sciences, Hainan Medical University Haikou 571199 PR China; 2 Department of Nuclear Medicine, the 928th Hospital of PLA Joint Logistics Force Haikou 570100 PR China; 3 Department of Hospital Infection Management and Public Health, Sanya Public Health Clinical Center Sanya Hainan 572022 PR China; 4 Department of Tropical Diseases, the Second Affiliated Hospital of Hainan Medical University Haikou 570100 PR China; 5 Department of Parasitology, Wenzhou Medical University Wenzhou 325035 PR China

**Keywords:** *Blastocystis*, Subtype, Prevalence, Domestic pig, Cattle, Hainan

## Abstract

*Blastocystis* is one of the most prevalent intestinal protozoans, transmitted through the fecal-oral route. Domestic pigs and cattle serve as important reservoirs for *Blastocystis*, playing a crucial role in its transmission dynamics. In the present study, a PCR-sequencing tool based on the small subunit ribosomal RNA (*SSU* rRNA) gene was employed to investigate the prevalence and subtypes of *Blastocystis* in 456 pig and 302 cattle fecal samples collected in Hainan, the only tropical island province in China. The overall prevalence of *Blastocystis* in pigs and cattle was 30.3% (138/456) and 13.2% (40/302), respectively. Six known subtypes-ST5 (*n* = 139), ST21 (*n* = 18), ST26 (*n* = 10), ST10 (*n* = 7), ST23 (*n* = 2), and ST25 (*n* = 2)-were identified, including 138 ST5 from pigs, and 18 ST21, 10 ST26, 7 ST10, 2 ST23, 2 ST25, and 1 ST5 from cattle. A novel ST5 sequence (OQ048307) from a pig and a novel ST10 sequence (OQ048308) from a cow were detected. Our results suggest that livestock may be an important potential reservoir for zoonotic *Blastocystis* infection in humans and provided reliable data for future research on subtype distribution and infection control of this protozoan in tropical regions.

## Abstract (fr)

*Blastocystis* est l’un des protozoaires intestinaux les plus répandus, transmis par voie oro-fécale. Les porcs et les bovins domestiques constituent d’importants réservoirs pour *Blastocystis* et jouent un rôle crucial dans la dynamique de sa transmission. Dans cette étude, une technique de séquençage par PCR basée sur le gène de l’ARN ribosomique de la petite sous-unité (ARNr 16S) a été utilisée pour étudier la prévalence et les sous-types de *Blastocystis* dans 456 échantillons fécaux de porcs et 302 échantillons fécaux de bovins prélevés à Hainan, la seule province insulaire tropicale de Chine. La prévalence globale de *Blastocystis* chez les porcs et les bovins était respectivement de 30,3% (138/456) et de 13,2% (40/302). Six sous-types connus – ST5 (n = 139), ST21 (n = 18), ST26 (n = 10), ST10 (n = 7), ST23 (n = 2) et ST25 (n = 2) – ont été identifiés, dont 138 ST5 chez le porc, 18 ST21, 10 ST26, 7 ST10, 2 ST23, 2 ST25 et 1 ST5 chez le bovin. Une nouvelle séquence ST5 (OQ048307) chez le porc et une nouvelle séquence ST10 (OQ048308) chez le bovin ont été détectées. Nos résultats suggèrent que le bétail pourrait constituer un important réservoir potentiel d’infections zoonotiques à *Blastocystis* chez l’homme et fournissent des données fiables pour les recherches futures sur la distribution des sous-types et la lutte contre ce protozoaire dans les régions tropicales.

## Introduction

*Blastocystis*, a widespread intestinal protozoan, colonizing both human and non-human hosts, is primarily transmitted through the fecal-oral route [60]. Recognized as a waterborne pathogen and prevalent eukaryotic organism, it is included in the World Health Organization (WHO) guidelines for drinking-water quality [70]. Although *Blastocystis* is frequently detected in asymptomatic individuals, its pathogenicity remains uncertain [7]. However, studies suggest that *Blastocystis* incubation can modulate the host immune response, which has been linked to irritable bowel syndrome [32]. Immunocompromised patients are particularly vulnerable to infection and associated symptoms [8].

Because it is impossible to distinguish different subtypes (STs) of *Blastocystis* by morphology, PCR-based molecular diagnostic methods have been used to analyze the sequence of the small subunit ribosomal RNA (*SSU* rRNA), thereby revealing its genetic diversity and potential transmission routes [72]. Currently, more than 40 subtypes have been identified in humans and animals [53, 61]. Seventeen subtypes of *Blastocystis* (ST1–ST10, ST12–ST14, ST16, ST23, ST35, and ST41) have been reported in humans [47, 53]. Among these, ST1–ST4 are the most common colonizers of the human intestinal tract, accounting for nearly 95% of *Blastocystis* isolates worldwide [47, 60]. These subtypes have also been detected in other mammals and birds [27], suggesting that domestic animals may serve as natural hosts for *Blastocystis* [63]. ST5, ST10, and ST14 are most frequently found in hoofed animals, including pigs, cattle, and sheep [12, 40, 47]. Meanwhile, ST1, ST3, ST5, and ST7 have been identified in both humans and domestic animals (pigs and cattle) in household settings [5, 16]. The overlap of subtypes suggests potential zoonotic transmission, highlighting the role of infected animals as a possible risk factor for human infections [5, 57, 58].

*Blastocystis* has been reported in domestic pigs and cattle worldwide, with overall prevalence rates ranging from 43.3% to 66.6% (Table S1) and 4.3% to 45.5% (Table S2), respectively. Available data indicate that pigs harbor nine distinct subtypes (ST1–ST5, ST7, ST10, ST14, and ST15), with ST5 being the predominant subtype (Table S1). In cattle, 23 subtypes (ST1–ST7, ST10, ST12–ST15, ST17, ST21, ST23–ST26, ST30, ST32, and ST42–ST44) have been detected, among which ST10 is the most prevalent globally (Table S2). In China, *Blastocystis* has been identified in livestock across multiple provinces, including Heilongjiang [16, 20, 64, 68], Hebei [61], Henan [23], Shaanxi [59], Shanxi [69, 71], Qinghai [50], Anhui [24], Jiangxi [55, 65], Hunan [65], Fujian [38, 65], Zhejiang, Guangdong, and Yunnan provinces [26, 76], as well as in the Xinjiang Uygur [66] and Guangxi Zhuang Autonomous Region [74]. The overall prevalence of *Blastocystis* in domestic pigs and cattle in China is 38.8% and 14.8%, respectively (Tables S1 and S2). Hainan is the only tropical island province in China, with a unique tropical climate and environment that are distinct from those of the provinces studied previously. Given that these unique conditions may influence the prevalence, genetic diversity, and transmission dynamics of *Blastocystis*, while data on the parasite in local livestock remain limited, this study was conducted. We collected 456 pig and 302 cattle samples from Hainan and amplified the *SSU* rRNA genes to investigate the prevalence of *Blastocystis* and assess its potential public health risk.

## Materials and methods

### Ethics statement

The sampling strategy in this study was implemented after obtaining written informed consent from farm owners for animal use. The study did not involve hunting, euthanasia procedures, or experiments on live vertebrates. All methods were conducted in accordance with relevant guidelines and regulations of the Ethics Committee of Hainan Medical University (approval no. HYLL-2022-405) and were reported following the ARRIVE guidelines (https://arriveguidelines.org).

### Specimen collection

**Figure 1:**
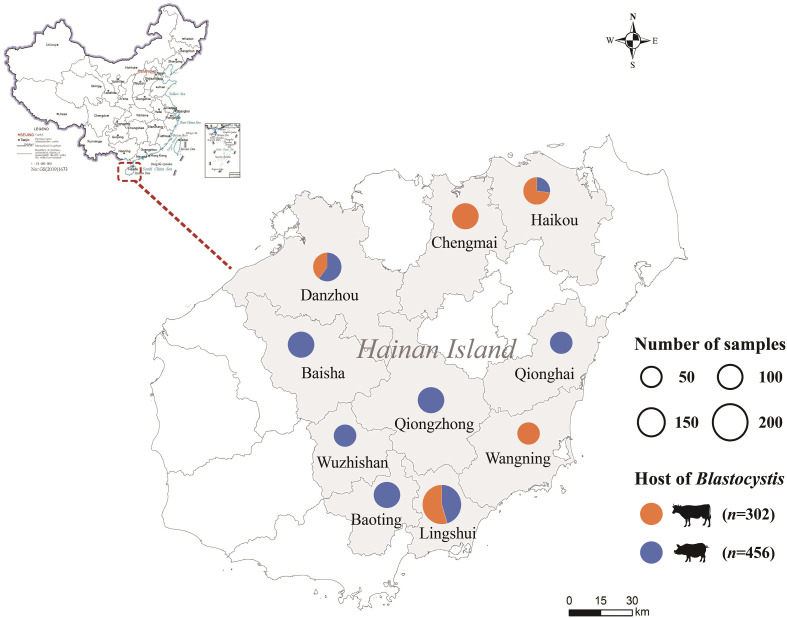
Map of sampling regions of domestic pigs and cattle in Hainan, China.

A total of 758 fresh fecal samples were collected from domestic pigs (*Sus scrofa* f. *domestica*) (*n* = 456) and cattle (*Bos taurus*) (*n* = 302) on farms across ten regions of Hainan Province, China, between March 2021 to October 2022 (Fig. 1). All sampled farms were large-scale breeding facilities complying with sanitary and production standards, housing 100–500 animals each. On each farm, specimens were collected from 20–30% of the total animals, all of which were healthy and showed no digestive symptoms. Fecal samples were preserved in individual sterile 50 mL centrifuge tubes with records of species, region and collection time. All the samples were transported under low-temperature and stored at −80 °C until analysis.

### DNA extraction and PCR amplification

The stored fecal samples were washed three times with distilled water, with each wash followed by centrifugation at 3000× *g* for 10 min. Genomic DNA was extracted from 200 mg of each processed fecal specimen using a QIAamp DNA stool mini kit (QIAGEN, Hilden, Germany), following the manufacturer’s protocol with modification of an increased lysis temperature (95 °C) to ensure optimal DNA yield. The extracted DNA was stored at −20 °C until PCR analysis.

The specimens were examined for *Blastocystis* by nested PCR amplification of the small subunit (*SSU*) rRNA gene using specific primers: forward primer RD1 (5′–GGAGGTAGTGAC AATAAATC–3′) and reverse primer RD2 (5′–TGCTTTCGCACTTGTTCATC–3′) [13], which yield a ~500 bp amplicon. PCR reactions were performed in a 25 μL volume using TaKaRa Taq DNA Polymerase (TaKaRa Bio Inc., Tokyo, Japan). The thermal cycling protocol consisted of: initial denaturation at 95 °C for 4 min; 35 cycles of denaturation at 95 °C for 30 s, annealing at 54 °C for 30 s, and extension at 72 °C for 30 s; followed by a final extension at 72 °C for 5 min. Each PCR run included two positive controls (*Blastocystis*-positive samples previously sequenced in our studies) and a negative control (reagent-grade water without template DNA). PCR products were electrophoresed on 1.5% agarose gels and visualized using GoldenView nucleic acid stain.

### Sequencing and phylogenetic analysis

All the *Blastocystis*-positive specimens were subjected to bidirectional sequencing. PCR products yielding novel sequences were additionally sequenced. Sequence data were processed and aligned using Clustal X v2.1 tools (http://www.clustal.org/). *Blastocystis* subtypes were determined through comparison with reference sequences in GenBank using the Basic Local Alignment Search Tool (BLAST) (http://www.ncbi.nlm.nih.gov/BLAST/). Phylogenetic analysis was conducted using MEGA 11 (Molecular Evolutionary Genetics Analysis Version 11.0) [62], employing both Neighbor-Joining (NJ) and Maximum Likelihood (ML) methods. Evolutionary distances were calculated using the Kimura 2-parameter model with 1,000 bootstrap replicates. The phylogenetic tree was rooted using Proteromonas lacertae (U37108) as the outgroup. Representative nucleotide sequences were submitted to GenBank under accession numbers: OQ727477–OQ727487, OQ727489–OQ727501, OQ048307, and OQ048308.

### Statistical analysis

Statistical analyses were performed using Statistical Package for the Social Sciences (SPSS) version 22.0 (SPSS Inc., Chicago, IL, USA). Fisher’s exact test was employed to compare *Blastocystis* prevalence across different regions groups, with statistical significance set at *p* < 0.05. Multilevel logistic mixed regression models were constructed to calculate odds ratios (OR) and 95% confidence intervals (CI), using *Blastocystis* prevalence and subtypes (STs) as primary outcome variables.

## Results

### Prevalence of *Blastocystis*

In this study, we collected 456 domestic pig and 302 cattle samples from Hainan Province. The overall prevalence of *Blastocystis* in pigs was 30.26% (138/456; 95% CI: 26.05–34.48), with regional prevalence rates ranging from 0% to 83.33% across eight sampling locations. Among cattle, the overall infection rate was 13.25% (40/302; 95% CI: 9.38–17.02), with regional prevalence ranging from 4.88% to 25.81% (Table 1).

**Table 1:** Risk factors associated with prevalence and subtype distribution of *Blastocystis* in domestic pigs and cattle in Hainan, China.

Hosts	Variable		Prevalence (%) (No. of positive / No. of examined) (95% CI)	OR (95% CI)	*p*-value	Subtype (*n*)
Domestic pig	Region	Baisha	16.8 (17/101) (9.51–24.09)	1.73 (1.60–1.87)	*p* < 0.001	ST5 (17)
		Baoting	83.3 (50/60) (73.86–92.74)	7.95 (5.83–10.84)		ST5 (50)
		Danzhou	43.1 (25/58) (30.36–55.84)	3.24 (2.82–3.71)		ST5 (25)
		Haikou	45.0 (9/20) (23.20–66.80)	5.53 (4.95–6.18)		ST5 (9)
		Lingshui	13.8 (11/80) (6.24–21.36)	Reference		ST5 (11)
		Qionghai	0 (0/30)	–		–
		Qiongzhong	0 (0/71)	–		–
		Wuzhishan	72.2 (26/36) (57.56–86.84)	7.22 (5.80–8.98)		ST5 (26)
		Total	30.3 (138/456) (26.08–34.52)			ST5 (138)
Cattle	Region	Chengmai	4.9 (4/82) (0.23–9.57)	Reference	*p* < 0.05	ST21 (3), ST26 (1)
		Danzhou	15.4 (6/39) (4.07–26.73)	2.48 (2.31–2.66)		ST21 (4), ST5 (1), ST26 (1)
		Haikou	14.8 (8/54) (5.33–24.27)	2.36 (2.20–2.54)		ST21 (3), ST10 (2), ST26 (2), ST23 (1)
		Lingshui	14.6 (14/96) (7.58–21.72)	2.19 (2.03–2.35)		ST21 (7), ST26 (5), ST10 (2)
		Wanning	25.8 (8/31) (10.40–41.20)	17.47 (13.72–22.24)		ST10 (3), ST25 (2), ST21 (1), ST23 (1), ST26 (1)
		Total	13.2 (40/302) (9.38–17.02)			ST21 (18), ST26 (10), ST10 (7), ST23 (2), ST25 (2), ST5 (1)

The “–” symbol indicates that data were not calculated, OR: Odd ratio, CI: Confidence interval.

### Subtype distribution of *Blastocystis*

Of the 138 domestic pig samples from different regions that tested positive by nested PCR and sequence analysis, all were identified as ST5 in this study (Table 1). Among the 40 *Blastocystis* isolates from cattle, six known subtypes (ST5, ST10, ST21, ST23, ST25, and ST26) were identified, with no mixed subtype infections detected in this study. ST21, ST26, and ST10 were the predominant subtypes in cattle in Hainan, accounting for 45.0% (18/40), 25.0% (10/40), and 17.5% (7/40), respectively. The other three subtypes occurred only occasionally: ST23 (5.0%, 2/40), ST25 (5.0%, 2/40), and ST5 (2.5%, 1/40). However, among the 8 *Blastocystis* isolates from cattle in Wanning, ST10 was the most abundant subtype at 37.5% (3/8), while ST21 was the most common in other sampling regions. Moreover, ST21 was the most prevalent subtype in cattle throughout the year (Table 1).

### Phylogenetic and sequence analysis of *Blastocystis* subtypes

**Figure 2:**
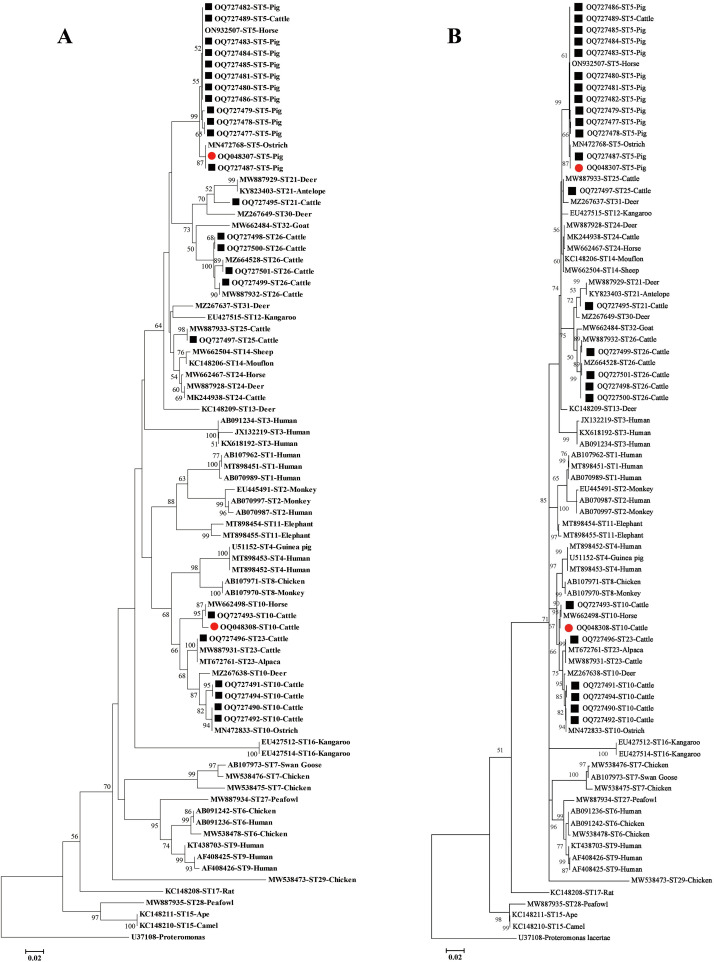
Phylogenetic analysis by Neighbor-Joining (A) and Maximum Likelihood (B) methods of *Blastocystis* isolates based on SSU rDNA gene sequences in this study. The known and novel sequences identified in this study are indicated by black squares and red circles, respectively. The sequence of *Proteromonas lacertae* (U37108) was as an outgroup.

**Figure 3:**
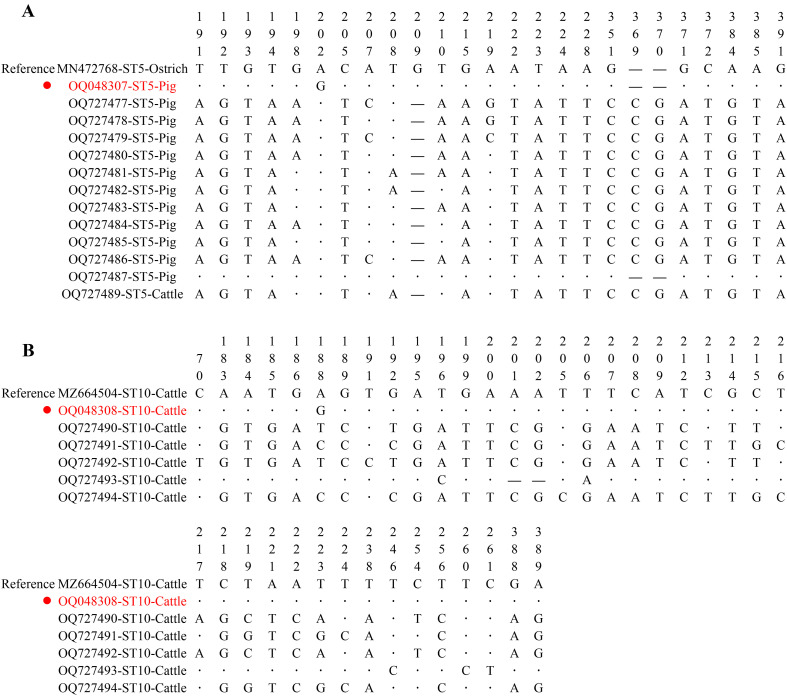
*SSU* rDNA gene sequence polymorphism among novel and known sequences of ST5 (A) and ST10 (B) with the reference sequence.

The results of the phylogenetic tree analysis by the Neighbor-Joining (NJ) and Maximum Likelihood (ML) methods showed consistent clustering patterns for *Blastocystis* subtypes, and the representative sequences of this study clustered with the reference sequences in their corresponding subtype branches (Figs. 2A and 2B). A nucleotide sequence with at least one nucleotide substitution, deletion, or insertion compared to known subtype sequences was considered a novel sequence. A novel sequence differing by at least 3% from others in a known clade may be defined as a new subtype [17]. The 176 sequences from *Blastocystis* isolates in this study have previously been described, except for a novel ST5 sequence (OQ048307) and a novel ST10 sequence (OQ048308). The 138 ST5 sequences from pigs (OQ727477–OQ727487 and OQ048307) and an ST5 sequence from cattle (OQ727489) clustered within the ST5 branch. A sequence alignment analysis of the ST5 sequences in this study showed that there were 25 polymorphic sites among them. The novel ST5 sequence (OQ048307) had 99.78% identity with MN472768 isolated from an ostrich in Brazil and a nucleotide substitution at position 202 (A → G) (Fig. 3A). The 7 ST10 sequences were isolated from cattle, OQ727490–OQ727492 and OQ727494 clustered as sister branches with MN472833 (Ostrich, Brazil), while OQ048308 and OQ727493 clustered as another sister branch with MW662498 (Horse, Colombia). The novel ST10 sequence (OQ048308) showed 99.79% identity to MZ664504 (Cattle, Spain) and contained an A → G substitution at position 188 (Fig. 3B). A total of 18 nucleotide sequences from cattle in the present study were identified as ST21 (OQ727495), displaying 100% identity with *Blastocystis* subtypes isolated from cattle in Spain (MZ664507). The 10 ST26 displayed 100% genetic identity with previously reported subtypes: OQ727498 (*n* = 3), OQ727499 (*n* = 2), OQ727500 (*n* = 4), and OQ727501 (*n* = 1) from cattle. ST23 (OQ727496, *n* = 2) and ST25 (OQ727497, *n* = 2) displayed an identity of 100% with a *Blastocystis* subtype isolated in a dairy heifer calve from the USA (MH634464) and a cow from Spain (MZ664506), respectively.

## Discussion

In the present study, *Blastocystis* was detected in 138 of 456 (30.3%) domestic pigs and 40 of 302 (13.2%) cattle through *SSU* rRNA gene PCR amplification. The overall prevalence in pigs was comparable to rates reported in Hunan, Jiangxi, and Fujian (31.4%, 394/1254) [65], but significantly higher than findings from Heilongjiang (8.8%, 6/68) [64], Shanxi (14.09%, 51/362) [69], and Xinjiang (21.7%, 174/801) [66] in China, Slovakia (12.0%, 12/100) [18], and the UK (16.7%, 2/12) [6]. However, it was lower than the pooled frequencies of 38.8% (China), 43.3% (Asia), and 46.8% (worldwide) reported in a meta-analysis encompassing more than 12,000 fecal samples globally (Table S1). The overall prevalence in Hainan cattle aligned with previous reports from Hebei (12.6%, 346/2746) [61], Shanxi (13.0%, 103/795) [71], Spain (13.3%, 118/890) [1, 48], and Egypt (13.9%, 78/563) [2, 41], but exceeded rates in America (4.3%, 112/2616) [21, 36, 54] and Korea (6.7%, 101/1512) [34]. These figures were nevertheless lower than the meta-analysis estimates for China (14.8%), Asia (16.9%), and the worldwide (15.4%) data from more than 18,000 samples (Table S2). Evidently, the prevalence of *Blastocystis* in pigs in regions south of the Changjiang River in China (e.g., Hainan, Hunan, Jiangxi, and Fujian) is higher than in regions north of the river. Beyond geographic and climatic influences, prevalence variations may reflect differences in environmental sanitation, production systems, detection methodologies, and livestock breed susceptibility [9, 51, 52, 65]. Further research is warranted to elucidate the factors driving this epidemiological variability.

The phylogenetic analysis demonstrated that all six known subtypes (ST5, ST10, ST21, ST23, ST25, and ST26) clustered within their respective subtype branches (Fig. 2). ST5, naturally hosted by hoofed animals including pigs, cattle, sheep, and camels, was the sole subtype detected in Hainan’s domestic pigs and was also found in one cattle specimen (Table 1). This finding aligns with global data from 54 surveys across 26 countries involving more than 5,600 *Blastocystis*-positive samples, where ST5 emerged as the predominant subtype in domestic pigs worldwide, particularly across Southeast Asian countries, including Cambodia [67], Indonesia [73], the Philippines [3], Thailand [46, 63], and Vietnam [6] (Table S1). A large number of studies have documented ST5 in cattle from various regions, including the United States [36, 54], Iran [39, 58], Malaysia [29, 49], and Türkiye [44] (Table S2). Although ST5 infections are considered rare in humans, cases have been reported in China [22, 75], Argentina [5], Cambodia [67], Türkiye [11], Malaysia [33], Syria [19] and Thailand [46]. These infections occur predominantly among individuals who cohabit with domestic animals [5] or work on commercial intensive pig farms [46, 67], suggesting zoonotic transmission through close animal contact or exposure to waste products from infected animals [15, 45, 46]. In our study, ST10 and ST23 were identified in 7 and 2 cattle specimens, respectively. ST10 represents the most prevalent subtype in cattle globally, as evidenced by meta-analysis of more than 18,000 samples from 25 countries (Table S2). While ST10 was initially considered primarily herbivore-associated with minimal reported human infections [56], it has since been documented in human populations in Egypt [41], Senegal [31], Vietnam [42], and Thailand [28]. Similarly, ST23 was identified as the dominant subtype in 12 human cases in Thailand [28], indicating its zoonotic potential and public health significance. Therefore, in subsequent studies, we will focus on investigating the colonization of zoonotic *Blastocystis* subtypes (particularly ST5, ST10, and ST23) among farm workers and in environmental samples (soil and waste water) in Hainan, while analyzing their transmission routes and assessing potential public health risks.

ST21, though uncommon in cattle worldwide (Table S2), showed unexpectedly high prevalence in Hainan cattle, consistent with findings from northern Spain [1]. ST26, another predominant subtype in Hainan cattle, has been frequently reported in Bangladesh [30], China [16, 68, 71] Colombia [4], and Spain [1]. ST25, detected in two Hainan cattle, appears sporadically in Malaysia [49], Portugal [25], Spain [1], Türkiye [14], and northeastern China [20]. Notably, ST21, ST25, and ST26 are considered non-zoonotic subtypes [14] and have not been identified in human infections.

Furthermore, this study identified and characterized two novel sequences: an ST5 variant (OQ048307) from pigs showing 99.78% identity with MN472768 (isolated from an ostrich in Brazil) [37] and an ST10 variant (OQ048308) from cattle demonstrating 99.79% identity with MZ664504 (from Spanish cattle) [1] (Fig. 2A and 2B). These discoveries expand our understanding of *Blastocystis* genetic diversity. Significantly, all six identified subtypes (ST5, ST10, ST21, ST23, ST25, and ST26) have been detected in water sources globally [10, 35], suggesting their potential for waterborne transmission [43]. To better understand *Blastocystis* epidemiology and transmission dynamics, and to effectively address this public health concern, future studies should incorporate larger sample sizes, diverse sample types, and human epidemiological data.

## Conclusions

This study represents the first investigation of *Blastocystis* colonization and subtype distribution in domestic pigs and cattle in Hainan, China. The overall prevalence rates were 30.3% (138/456) in pigs and 13.2% (40/302) in cattle. Six known subtypes (ST5, ST10, ST21, ST23, ST25, and ST26) were identified, along with two novel sequences: an ST5 variant (GenBank accession OQ048307) from pigs and an ST10 variant (OQ048308) from cattle. These findings provide valuable baseline data for future studies on *Blastocystis* epidemiology in domestic animals and inform infection control strategies in tropical regions.

## Data Availability

The datasets supporting the results of this article have been submitted to GenBank and accession numbers are shown in the article. All relevant data are within the article. The sequence data were submitted to the GenBank database under the accession numbers: OQ727477–OQ727487, OQ727489–OQ727501, OQ048307, and OQ048308.

## Supplementary materials

*Table S1*: The prevalence and subtypes distribution of *Blastocystis* in domestic pigs worldwide. *Table S2*: The prevalence and subtypes distribution of *Blastocystis* in cattle worldwide. The Supplementary material of this article is available at https://www.parasite-journal.org/10.1051/parasite/2025070/olm.
