# Supplementary material for: Molecular characterization and zoonotic potential of Blastocystis subtypes in domestic pigs and cattle from Hainan, a tropical island province in China
Source: Parasite. 2025 Dec 5;32:77. doi: 10.1051/parasite/2025070 (PMC12680373; doi:10.1051/parasite/2025070)
Supplement: Supplementary file 1 — Table S1: The prevalence and subtypes distribution of Blastocystis in domestic pigs worldwide. [file parasite-32-77-s1.pdf]

Table S1. The prevalence and subtypes distribution of *Blastocystis* in domestic pigs worldwide.

Continent	Country	No. of positive	No. of examined	Infection rate (%)	Method	Subtype (n)	References
Asia	Cambodia	33	73	45.2	Mol	ST5 (20), ST5/Unknown STs(13)	[86]
	China	2461	6338	38.8	Mol	ST5(2000), ST1(142), ST3(87), ST14(18), ST1(1), Unknown STs(8)	This study, [16, 26, 30, 45, 73, 82-84, 88, 92, 95]
	India	86	91	94.5	Mic	--	[9, 74]
	Indonesia	81	93	87.1	Mol	ST5(14), ST1/ST2/ST5(31), ST1/ST5(12), ST2/ST5(8), ST5/ST7(8), Unidentified(8)	[91]
	Japan	148	201	73.6	Mic, Mol	ST5(31), ST5/ST1/ST3(26), ST5/ST1/ST2/ST3(10), ST5/ST1(8), ST5/ST1/ST2(2), ST5/ST2(1), Unidentified(70)	[3, 4, 42]
	Korea	390	646	60.4	Mol	ST5(56), ST1(14), ST3(7), ST2(3), Unidentified(310)	[52]
	Nepal	4	11	36.4	Mol	ST4(2), Unidentified(2)	[39]
	Philippines	12	12	100	Mol	ST5(5), ST3(3), ST2(2), ST1(1), ST3/ST5/ST7(1)	[6]
	Thailand	116	241	48.1	Mic, Mol	ST5(76), ST1(18), ST3(1), ST15(1), Unidentified(20)	[44, 54, 64, 79-81]
	Vietnam	12	12	100	Mol	ST5 (12)	[8]
	Subtotal	3343	7718	43.3		ST5(2214), ST1(175), ST3(98), ST14(18), ST2(5), ST4(2), ST10(1), ST15(1), Mixed STs(120), Unknown STs (8), Unidentified(410)	
Europe	Bohemia	418	576	72.6	Mic	--	[53]
	Czech Republic	344	416	82.7	Mic	--	[53]
	Denmark	193	259	75.0	Mol	ST5(85), ST1(8), ST15(5), ST3(2), ST5/ST1(59), ST5/ST15(26), ST5/ST3(2), ST1/ST15(2), ST1/ST5/ST15(2), ST1/ST3/ST5(1), ST3/ST5/ST15(1)	[76]
	United Kingdom (UK)	2	12	16.7	Mol	ST5(1), Unknown STs(1)	[8]

Table S1. (Continued)

Continent	Country	No. of positive	No. of examined	Infection rate (%)	Method	Subtype (n)	References
Europe	Germany	34	41	82.9	Mol	ST5(12), ST1(1), ST5/ST15(9), ST1/ST3/ST5(4), ST1/ST5/ST15(3), ST1/ST5(2), ST2/ST5/ST15(1), ST1/ST2/ST3/ST5(1), ST1/ST3/ST5/ST15(1)	[37, 89]
	Italy	36	50	72.0	Mol	ST5(24), ST3(2), ST1(1), ST5/ST15(3), ST3/ST5(1), Unidentified(5)	[25, 66]
	Poland	57	149	38.3	Mol	ST5(51), ST1(1), ST5/ST1(3), ST5/ST3(1), ST3/ST1(1)	[65]
	Serbia	39	48	81.3	Mic, Mol	ST5(39)	[67, 68]
	Slovakia	12	100	12.0	Mol	ST5 (12)	[17]
	Spain	553	1416	73.1	Mic, Mol	ST5(316), ST1(24), ST3(23)	[18, 49, 56, 61]
	Subtotal	1688	3067	55.0		ST5(540), ST1(35), ST3(27), ST15(5), Mixed STs(114), Unknown STs(1), Unidentified(5)	
North America	United States of America (U.S.A.)	20	41	48.8	Mol, Sero	ST5(5), Unidentified(6)	[20, 58]
	Subtotal	20	41	48.8		ST5(5), Unidentified(6)	
Oceania	Australia	389	584	66.6	Mic, Mol	ST5(347), ST1(3), ST3(2), ST5/ST1(6), ST5/ST3(4), ST5/ST1/ST3(3), ST5/Unknown STs(4), ST5/ST1/ST3(7), ST5/ST1/ST3(13)	[13, 62, 85, 86]
	Subtotal	389	584	66.6		ST5(347), ST1(3), ST3(2), Mixed STs(37)	
South America	Argentina	19	42	45.2	Mic	--	[7]
	Brazil	50	80	62.5	Mic, Mol	ST1(4), ST5(2), ST3(1), Unidentified(10)	[14, 23, 93]

	Colombia	52	74	70.3	Mol	ST5(24), ST3(2), ST15(1), ST1/ST5(7), ST5/ST15(4), ST3/ST5(1), ST3/ST5/ST15(3), ST1/ST3/ST5(5)	[33, 34]

Table S1. (Continued)

Continent	Country	No. of positive	No. of examined	Infection rate (%)	Method	Subtype (n)	References
South America	Ecuador	13	26	50.0	Mic	--	[28]
	Subtotal	134	222	60.4		ST5(26), ST1(4), ST3(3), ST15(1), Mixed STs(20), Unidentified(10)	
	Total	5574	11632	47.9		ST5(3132), ST1(217), ST3(130), ST14(18), ST15(7), ST2(5), ST4(2), ST10(1), Mixed STs(291), Unknown STs(10), Unidentified(431)	

“Mic” indicates microscopic detection method.

“Mol” indicates molecular detection method.

“Sero” indicates serological detection method.

“Unknown STs” indicates samples that were successfully amplified by PCR and sequenced but were identified as unknown subtypes.

“Mixed STs” indicates the detection of two or more subtypes within a single positive sample.

“Unidentified’’ indicates positive samples without sequencing or with failed sequencing.

“--” indicates samples that tested positive by microscopy or serological detection method, but were not subjected to molecular identification.
