# Supplementary material for: Molecular characterization and zoonotic potential of Blastocystis subtypes in domestic pigs and cattle from Hainan, a tropical island province in China
Source: Parasite. 2025 Dec 5;32:77. doi: 10.1051/parasite/2025070 (PMC12680373; doi:10.1051/parasite/2025070)
Supplement: Supplementary file 2 — Table S2: The prevalence and subtypes distribution of Blastocystis in cattle worldwide. [file parasite-32-77-s2.pdf]

Table S2. The prevalence and subtypes distribution of *Blastocystis* in cattle worldwide.

Continent	Country	No. of positive	No. of examined	Infection rate (%)	Method	Subtype (n)	References
Africa	Algeria	2	2	100	Mic, Mol	ST6(2)	[11]
	Egypt	78	563	13.9	Mic, Mol	ST14(15), ST10(14), ST4(2), ST3(1), ST10/ST14(16), Unidentified(30)	[2, 48]
	Subtotal	80	565	14.1		ST14(15), ST10(14), ST4(2), ST6(2), ST3(1), Mixed STs(16), Unidentified(30)	
Asia	Bangladesh	103	699	14.7	Mol	ST10(57), ST26(14), ST24(13), ST4(5), ST21(5), ST3(4), ST25(3), ST14(2)	[36]
	China	1272	8569	14.8	Mol	ST10(709), ST14(219), ST5(119), ST26(59), ST1(52), ST12(38), ST21(27), ST3(21), ST25(6), ST23(3), ST24(3), ST2(2), ST4(2), ST10/ST25(1), ST10/ST26(1), ST10/ST23/ST25(1), Unknown STs(6)	This study, [16, 19, 24, 40, 60, 77, 82, 87, 90, 94]
	Indonesia	214	608	35.2	Mic, Mol	ST10(20)	[31, 78]
	Iran	131	675	19.4	Mic, Mol	ST10(31), ST5(19), ST14(17), ST3(8), ST1(5), ST6(2), ST7(1), ST3/ST5(2), Unidentified(24)	[12, 32, 47, 63, 69, 71, 72]
	Japan	121	227	53.3	Mic, Mol	ST14(44), ST10(1), Unidentified(27)	[3, 4, 43]
	Korea	101	1512	6.7	Mol	ST14(10), ST10(9), ST1(6), ST5(5)	[38]
	Lebanon	161	254	63.4	Mol	ST10(55), ST14(46), ST2(10), ST1(9), ST5(3), ST3(1), ST7(1), Mixed STs(36)	[29]
	Malaysia	56	250	22.4	Mol	ST10(18), ST5(9), ST14(8), ST3(6), ST1(2), ST4(2), ST25(1), Unidentified(10)	[35, 46, 59]
	Nepal	1	6	16.7	Mol	ST6(1)	[39]
	Thailand	24	67	35.8	Mol	ST12(4), ST10(2), ST23(1), ST14/ST24/ST25(2), Unknown STs(15)	[79, 81]
	United	5	22	22.7	Mol	ST10(3), Unknown STs(2)	[5]

	Arab Emirates						
	Turkey	69	441	15.6	Mol	ST10(46), ST14(8), ST5(2), ST12(2), ST1(1), ST3(1), ST13(1), ST25(1), Unidentified(7)	[10, 15, 50, 51]

Table S2. (Continued)

Continent	Country	No. of positive	No. of examined	Infection rate (%)	Method	Subtype (n)	References
Asia	Subtotal	2258	13330	16.9		ST10(951), ST14(354), ST5(157), ST1(75), ST26(73), ST12(44), ST3(41), ST21(32), ST24(16), ST2(12), ST25(11), ST4(9), ST23(4), ST6(3), ST7(2), ST13(1), Mixed STs(43), Unknown STs(23), Unidentified(68)	
Europe	Denmark	25	25	100	Mol	ST10(22), ST5(3)	[75]
	United Kingdom (UK)	7	31	22.6	Mol	ST10(3), ST1(1), ST5(1), Mixed STs(2)	[8]
	France	0	2	0	Mol	--	[8]
	Germany	10	66	15.2	Sero	--	[37]
	Italy	27	53	50.9	Mol	ST5(19), ST3(2), ST1(1), ST5/ST15(3), ST3/ST5(1), Unknown STs(1)	[8, 25, 66]
	Portugal	28	87	32.2	Mol	ST23(1), Mixed STs(27)(Mixed STs including ST1, ST5, ST10a, ST10b, ST13, ST14, ST21, ST23, ST24a, ST24b, ST24c, ST25, ST26, ST30, ST42a, ST42b, ST43, ST44)	[22, 27]
	Spain	118	890	13.3	Mic, Mol	ST10/ST2/ST26(15), ST10/ST14/ST21 /ST25/ST26(15), ST10/ST14/ST21/ST23/ST25/ST26(12), ST10/ST21/ST23/ST25/ST26(8), ST10/ST14/ST21/ST23/ST24/ST25/ST26(7), ST10/ST14/ST25/ST26(5), ST10/ST14/ST21/ST24/ST25/ST26(5), ST10/ST21 /ST25/ST26(4), ST10/ST23/ST25/ST26(4), ST10/ST14/ST21/ST24/ST26(4), ST5/ ST10/ST14/ST21/ST25/ST26(4), ST5/ST10/ST25/ST26(3), ST10/ST14/ST21/ST23/ST24/ST26(3), ST10/ST25(1), ST25/ST26(1), ST5/ST10/ST25(1), ST5/ST10/ST14(1), ST10/ST14/ST24(2),	[1, 55]

						ST10/ST21/ST26(1), ST14/ST25/ST26(1), ST10/ST14/ST21/ST24(1), ST10/ST21/ST23/ST26(1), ST10/ST21/ST24/ST26(1), ST5/ST10/ST14/ST25/ST26(1), ST5/ST10/ST14/ST24/ST26(1),	

Table S2. (Continued)

Continent	Country	No. of positive	No. of examined	Infection rate (%)	Method	Subtype (n)	References
Europe	Spain	118	890	13.3	Mic, Mol	ST10/ST14/ST24/ST25/ST26(1), ST5/ST10/ST14/ST23/ST25/ST26(1), ST10/ST14/ST21/ST23/ST24/ST25(1), ST10/ST21/ST23/ST24/ST25/ST26(1), ST1/ST5/ST10/ST14/ST21/ST25/ST26(1), ST3/ST10/ST14/ST21/ST23/ST24/ST25/ST26(1)	[1, 55]
	Subtotal	215	1154	18.6		ST10(25), ST5(23), ST3(2), ST1(2), ST23(1), Mixed STs(139), Unknown STs(1)	
North America	America	112	2616	4.3	Mol	ST5(27), ST4(18), ST10(9), ST14(8), ST3(4), ST17(3), ST26(3), ST21(2), ST24(1), ST25(1), ST10/ST14/ST26(1), ST10/ST24(1), ST23/ST26(1), ST10/ST14(2), Unknown STs(1), Mixed STs(30)(Mixed STs including ST1–ST6, ST10a, ST10b, ST10c, ST10d, and ST10e, ST14, ST21, ST23–ST26 and one Unknown STs)	[21, 41, 70]
	Subtotal	112	2616	4.3		ST5(27), ST4(18), ST10(9), ST14(8), ST3(4), ST17(3), ST26(3), ST21(2), ST24(1), ST25(1), Unknown STs(1), Mixed STs(35)	
South America	Brazil	38	89	42.7	Mic, Mol	ST10(1), Unknown STs(6)	[14, 23, 93]
	Colombia	49	112	43.8	Mol	ST1(12), ST3(8), ST10(1), ST14(1), ST25(1), ST10/ST25(4), ST10/ST14/ST21/ST25/ST26(3), ST10/ST21/ST23/ST25/ST26(3), ST10/ST14(2), ST23/ST25/ST26(2), ST10/ST25/ST26(2), ST10/ST14/ST21/ST23/ST24/ST25/ST26/ST32(1), ST10/ST14/ST23/ST24/ST25/ST26(1), ST10/ST14/ST25(1), ST10/ST21/ST23/ST24/ST25/ST26(1), ST10/ST21/ST23/ST24/ST26(1),ST10/ST21/ST23/ST25(1),	[33, 34, 57]

Continent	Country	No. of positive	No. of examined	Infection rate (%)	Method	Subtype (n)	References
						ST10/ST21/ST25/ST26(1), ST10/ST23/ST25(1), ST10/ST10/ST26(1), ST5/ST10/ST26(1)	
	Ecuador	25	45	55.6	Mic	--	[28]
	Subtotal	112	246	45.5		ST1(12), ST3(8), ST10(2), ST14(1), ST25(1), Mixed STs(26), Unknown STs(6)	

Table S2. (Continued)

Continent	Country	No. of positive	No. of examined	Infection rate (%)	Method	Subtype (n)	References
	Total	2777	17911	15.5		ST10(1001), ST14(378), ST5(207), ST1(89), ST26(76), ST3(56), ST12(44), ST21(34), ST4(29), ST25(13), ST24(17), ST2(12), ST6(5), ST23(5), ST17(3), ST7(2), ST13(1), Mixed STs(259), Unknown STs(31), Unidentified(98)	

"Mic" indicates microscopic detection method.

"Mol" indicates molecular detection method.

"Sero" indicates serological detection method.

"Unknown STs" indicates samples that were successfully amplified by PCR and sequenced but were identified as unknown subtypes.

"Mixed STs" indicates the detection of two or more subtypes within a single positive sample.

"Unidentified'' indicates positive samples without sequencing or with failed sequencing.

"--" indicates samples that tested positive by microscopy or serological detection method, but were not subjected to molecular identification.
